# Profile and Determinants of Retinal Optical Intensity in Normal Eyes with Spectral Domain Optical Coherence Tomography

**DOI:** 10.1371/journal.pone.0148183

**Published:** 2016-02-10

**Authors:** Binyao Chen, Enting Gao, Haoyu Chen, Jianling Yang, Fei Shi, Ce Zheng, Weifang Zhu, Dehui Xiang, Xinjian Chen, Mingzhi Zhang

**Affiliations:** 1 Joint Shantou International Eye Center, Shantou University and the Chinese University of Hong Kong, Shantou, China; 2 School of Electronics and Information Engineering, Soochow University, Suzhou, China; Purdue University, UNITED STATES

## Abstract

**Purpose:**

To investigate the profile and determinants of retinal optical intensity in normal subjects using 3D spectral domain optical coherence tomography (SD OCT).

**Methods:**

A total of 231 eyes from 231 healthy subjects ranging in age from 18 to 80 years were included and underwent a 3D OCT scan. Forty-four eyes were randomly chosen to be scanned by two operators for reproducibility analysis. Distribution of optical intensity of each layer and regions specified by the Early Treatment of Diabetic Retinopathy Study (ETDRS) were investigated by analyzing the OCT raw data with our automatic graph-based algorithm. Univariate and multivariate analyses were performed between retinal optical intensity and sex, age, height, weight, spherical equivalent (SE), axial length, image quality, disc area and rim/disc area ratio (R/D area ratio).

**Results:**

For optical intensity measurements, the intraclass correlation coefficient of each layer ranged from 0.815 to 0.941, indicating good reproducibility. Optical intensity was lowest in the central area of retinal nerve fiber layer, ganglion cell layer, inner plexiform layer, inner nuclear layer, outer plexiform layer and photoreceptor layer, except for the retinal pigment epithelium (RPE). Optical intensity was positively correlated with image quality in all retinal layers (0.553<β<0.851, p<0.01), and negatively correlated with age in most retinal layers (-0.362<β<-0.179, p<0.01), except for the RPE (β = 0.456, p<0.01), outer nuclear layer and photoreceptor layer (p>0.05). There was no relationship between retinal optical intensity and sex, height, weight, SE, axial length, disc area and R/D area ratio.

**Conclusions:**

There was a specific pattern of distribution of retinal optical intensity in different regions. The optical intensity was affected by image quality and age. Image quality can be used as a reference for normalization. The effect of age needs to be taken into consideration when using OCT for diagnosis.

## Introduction

The retina plays an important role in visual sense of human. Information, such as morphology, thickness, as well as volume changes of the retina, provided by imaging techniques, would be of great value in the diagnosis and follow-up of retinal diseases [1–3]. High resolution cross section imaging of the retina, based on using optical coherence tomography (OCT) to measure the magnitude of backscattered light signals from the tissue [4], should lead to a better understanding of retinal microstructure *in vivo*. Retinal reflectivity alterations caused by pathological processes can be easily observed on OCT scans [5–7]. Although there are no algorithms analyzing tissue reflectivity available for commercial OCT instruments, advances in imaging analysis technology have allowed quantitative mapping of tissue optical intensity [8–13].

Optical intensity analysis is an established method used in biochemistry for semi-quantification of proteins [14], DNA and RNA [15], and has also been applied to assessment of bone mineral density [16] and skin fibrosis in systemic sclerosis [17]. In ophthalmology, optical intensity can provide clues for distinguishing pathological changes. The optical intensity of the retinal nerve fiber layer (RNFL) in glaucoma patients has been shown to be lower than that in normal subjects, and decreases with increasing disease severity [18, 19]. Compared to normal vitreous, exudation lesions show higher reflectivity, whereas degeneration changes have lower optical intensity [9]. In addition, reflectivity of the cystoid space varies with fluorescein pooling intensity, suggesting that blood—retinal barrier disruption can lead to content changes in diabetic macular edema [11]. Moreover, loss of reflectivity in the photoreceptor ellipsoid region has been reported to occur early and can be detected from the first clinical presentation in patients with idiopathic perifoveal telangiectasia [8]. A study by Giani et al., using OCT, shows that quantitative analysis of choroidal neovascularization (CNV) reflectivity can differentiate leaky CNV from that without leakage, providing additional information regarding the fluorescein angiography leakage status [10]. These studies suggest the possibility of using optical intensity in diagnosis and follow up of glaucoma and retinal diseases with OCT.

**Fig 1:**
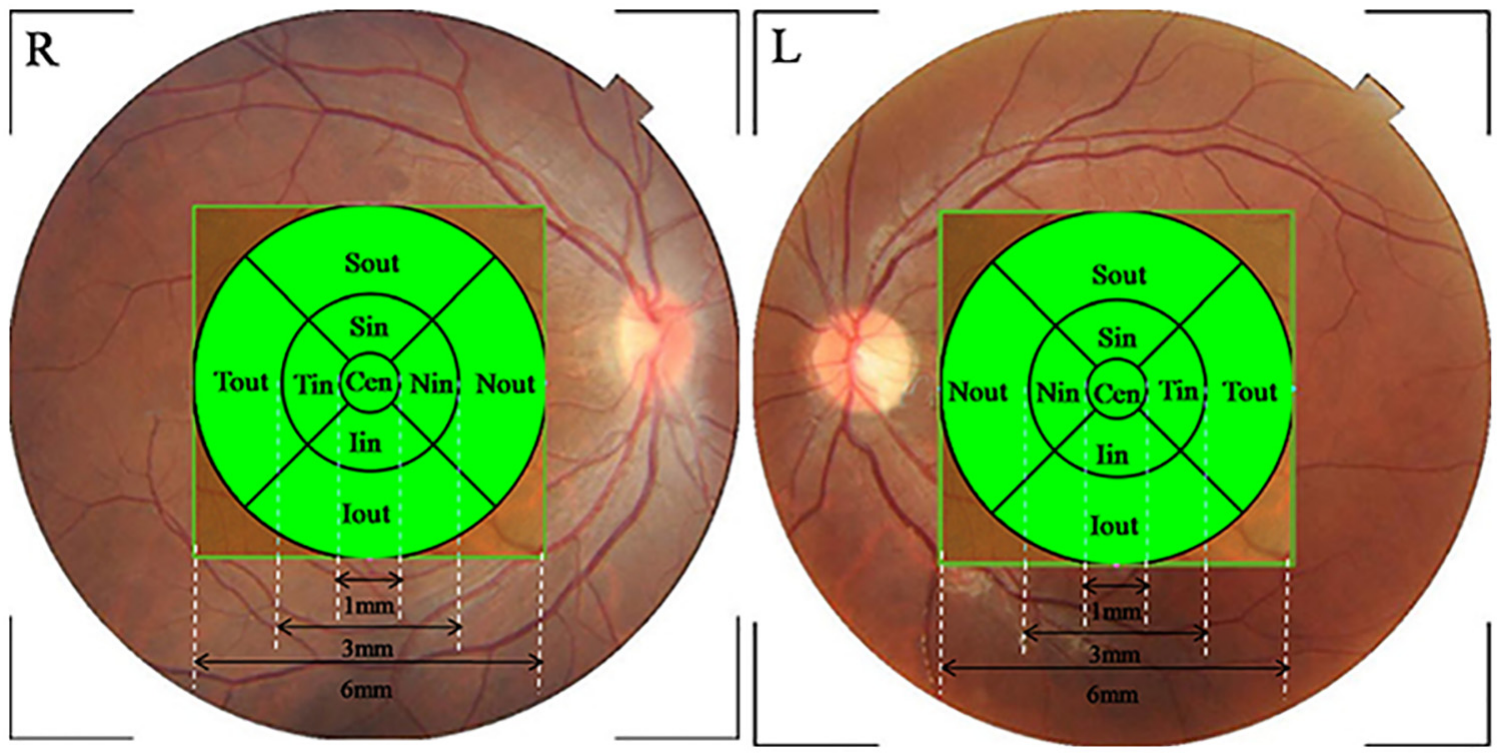
Early Treatment Diabetic Retinopathy Study (ETDRS) chart of the right (R) and left (L) eyes. The ETDRS plot is centered at the fovea. It includes three circles with diameters of 1 mm, 3 mm and 6 mm. The area is further divided into four quadrants: superior, inferior, nasal and temporal. Cen, central subfield; Sin, Superior inner ring; Nin, Nasal inner ring; Iin, Inferior inner ring; Tin, Temporal inner ring; Sout, Superior outer ring; Nout, Nasal outer ring; Iout, Inferior outer ring; Tout, Temporal outer ring.

Since application of newly developed parameters depends on an understanding of normal conditions, it is critical to establish a normative database of specific criteria. However, to our knowledge, few studies have been carried out on retinal optical intensity distribution in normal subjects. The effect of determinants such as sex, age, race, optic disc area, axial length and refractive error [20–23] which affect retinal thickness measurements on optical intensity remained unknown. In the present study, we investigated the retinal optical intensity distribution, in each retinal layer and nine macular sectors based on areas defined in the Early Treatment Diabetic Retinopathy Study (ETDRS) [24] (Fig 1), by analyzing three-dimensional data of normal subjects in different age groups with our graph-based algorithm [25, 26]. To collect reference data on the determinants, we also evaluated the effects of age, sex, height, weight, refractive status, axial length, image quality, optic disc area and rim/disc area ratio on optical intensity.

## Subjects and Method

### Subjects

Two hundred fifty-seven healthy volunteers were recruited at the Joint Shantou International Eye Center (JSIEC) in China between September 2013 and November 2013. This study was approved by the Institutional Review Board of JSIEC, Shantou University and the Chinese University of Hong Kong and followed the tenets of the Declaration of Helsinki. The methods were carried out in accordance with the approved guidelines. All participants engaged in an informed consent process and signed a written consent document before the study procedures were initiated.

General information, such as gender, age, medical history, height and weight, were recorded. All participants underwent comprehensive ophthalmologic examinations, including uncorrected visual acuity, best-corrected visual acuity (BCVA) testing with a logarithmic visual acuity chart, refractive error, non-contact tonometry, slit-lamp biomicroscopy, fundus examination, a Humphrey visual field (VF) test and an OCT scan. The inclusion criteria consisted of the following: (1) age older than 18 years old, (2) no history of glaucoma, retinal disease or diabetic mellitus, (3) a BCVA of 0.3 LogMAR or better, with spherical refraction between -6.0 to 6.0 diopters, (4) intraocular pressure of 21 mmHg or less, (5) no retinal pathologic features, and (6) a normal VF. One eye was randomly chosen if both eyes were eligible. Exclusion criteria were as follows: a VF defect or pathological retinal changes under OCT examination. For the VF test, the fixation losses should be <20% with false-positive & false-negative rates <15%. Criteria for a VF defect include: (1) three or more significant (P<0.05) continuous points with at least one at the P<0.01 level in a single hemifield in the pattern deviation plot, (2) glaucoma hemifield test outside normal limits, and (3) a pattern standard deviation significantly elevated beyond the 5% level[27].

### OCT imaging

All subjects received scans by experienced operators (B.C and J.Y.) with Topcon 3D OCT-2000 (Topcon, Tokyo, Japan, software version: 8.11.003.04) without pupil dilatation. Three-dimensional image data were acquired using the scan mode of 3D macular (512X128) centered at the fovea and covering a 6X6 mm^2^ area. Scanning was performed with the measurement beam perpendicular to the retina (light entered the eyes across the central position of the pupil). Forty-four eyes were randomly chosen to be scanned by the two operators on the same day for reproducibility analysis. The axial resolution was 5–6 μm and transverse resolution was 20 μm. Images, of a quality of 45 or higher, were included. Parameters, such as disc area and rim/disc area ratio, were obtained through the 3D disc (512X128) scan. Reference plane for optic disc parameters analysis was set at 120 μm above the RPE according to the default setting.

### OCT raw data analysis

**Fig 2:**
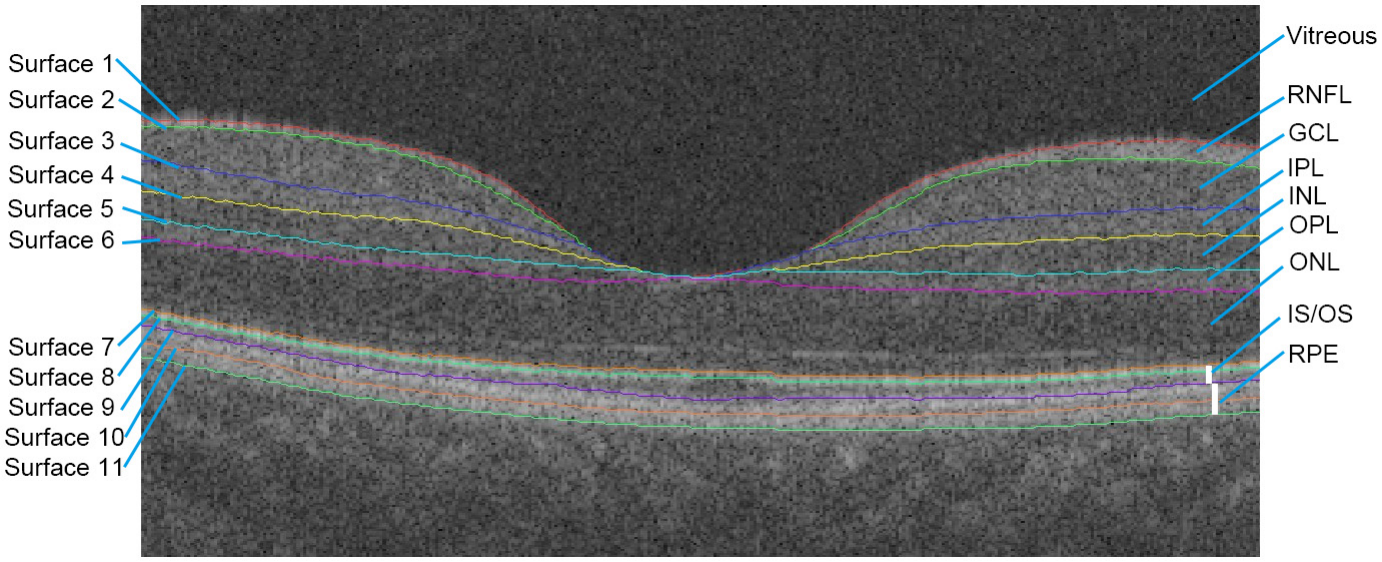
Intraretinal segmentation by the graph-based algorithm. Eleven intraretinal surfaces were detected automatically with our algorithm. Retinal layers could be identified as the following: RNFL, GCL, IPL, INL, OPL, ONL, photoreceptor layer and RPE.

Three-dimensional raw data exported from the OCT device in “fds.” format, containing 512x128x885 voxels, were imported into our automatic graph search algorithm which had good performance in retinal layer segmentation and thickness analysis as compared to the built-in Topcon OCT algorithm [25, 26]. The data analysis workflow included three steps: preprocessing, layer segmentation and optical intensity analysis. The preprocessing part was a denoising step. OCT data speckle noise was first reduced by a curvature anisotropic diffusion filter. Retinal boundaries were later automatically detected by finding an optimal closed set in a vertex-weighted graph [25]. The RNFL, ganglion cell layer (GCL), inner plexiform layer (IPL), inner nuclear layer (INL), outer plexiform layer (OPL), outer nuclear layer (ONL), photoreceptor layer and retinal pigment epithelium (RPE) were identified (Fig 2). Every B-scan image was visually inspected by an ophthalmologist (B.C.) and excluded if any misidentification of boundaries between retinal layers occurred. Subsequently, information from surface 1 to surface 11 were utilized. In this part, we identified the lowest location of surface 1 (Fig 2), built an ETDRS chart centered at this point and measured the level of gray of every voxel in each layer and each ETDRS sector. Optical intensity was calculated as the mean gray value of all voxels in the target area. Since all of the raw scan data were exported as 16-bit gray-scale images, the gray-scale value of voxels ranged from 0 to 65535. Arbitrary units (AU) were used instead of decibels as raw data were employed.

### Statistical analysis

Statistical analysis was performed with commercial statistical software (IBM SPSS Statistics v. 17 for Windows; SPSS Inc. Chicago, IL). To evaluate interoperator differences, the intraclass correlation coefficient (ICC) was used. The mean and standard deviation (SD) of optical intensity for each retinal layer were calculated in the six age groups. Independent samples t-test and Pearson’s correlation were used to evaluate the effect of sex, age, height, weight, spherical equivalent, axial length, image quality, disc area and rim/disc area ratio on optical intensity. Factors significant at P<0.05 were included in the stepwise multiple regression analysis. Sigmaplot (version 12.5, Systat Inc.) was employed to draw contour plots. A P<0.05 was considered statistically significant.

## Results

### Demographic and Ocular Characteristics of Study Participants

Two hundred fifty-seven subjects were recruited. Among them, 26 (10.1%) were excluded because of VF defect (4.7%) and boundary misidentification (5.5%), resulting in a total of 231 eyes, from 102 (44.2%) males and 129 (55.8%) females, being included in the study. Participants were divided into six groups according to age (Table 1). Demographic and ocular features are presented in Table 2.

**Table 1:** Distribution of age groups.

Group	Age(year)	Male	Female	Total
**1**	20–29	15	30	45 (19.5%)
**2**	30–39	22	24	46 (19.9%)
**3**	40–49	16	20	36 (15.6%)
**4**	50–59	18	22	40 (17.3%)
**5**	60–69	16	17	33 (14.3%)
**6**	70+	15	16	31 (13.4%)
**Total**	—	102	129	231 (100%)

**Table 2:** Characteristics of subjects.

	Male	Female	Total
**L/R**	55/47	63/66	118/113
**Age (y)**	48.22 ± 16.50	45.87 ± 17.15	46.90 ± 16.87
**Height (cm)**	168.99 ± 5.40	156.98 ± 4.88	162.28 ± 7.86
**Weight (kg)**	66.36 ± 9.20	54.84 ± 9.31	60.30 ± 12.96
**SE refraction (D)**	-0.61 ± 1.67	-0.71 ± 2.08	-0.67 ± 1.91
**Axial Length (mm)**	23.88 ± 1.03	23.40 ± 1.11	23.65 ± 1.22
**IOP (mmHg)**	13.53 ± 2.87	14.15 ± 2.75	13.87 ± 2.82
**MD (dB)**	-1.34 ± 1.32	-1.27 ± 1.35	-1.30 ± 1.33
**PSD (dB)**	1.49 ± 0.29	1.65 ± 0.68	1.58 ± 0.55
**ImageQ**	57.94 ± 4.47	58.40 ± 4.33	58.19 ± 4.39
**Disc area (mm**^**2**^**)**	2.30 ± 0.45	2.25 ± 0.37	2.27 ± 0.41
**R/D area ratio**	0.64 ± 0.21	0.68 ± 0.20	0.66 ± 0.21

L/R: left/right eye; SE: spherical equivalent; IOP: intraocular pressure; MD: mean deviation; PSD: pattern standard deviation; ImageQ: image quality; R/D area ratio: rim/disc area ratio

### Retinal optical intensity measurement

Table 3 shows the reproducibility of retinal optical intensity analysis. For measurements over the entire scan area and each retinal layer, ICCs ranged from 0.815 to 0.941.

**Table 3:** Reproducibility of retinal optical intensity analysis.

Measurement	Operator A	Operator B	ICC value	P value
**Image Q**	56.89	56.84	0.844	<0.01
**RNFL**	31009.16	31077.32	0.892	<0.01
**GCL**	26291.55	26326.66	0.815	<0.01
**IPL**	26345.36	26384.82	0.838	<0.01
**INL**	23247.75	23303.70	0.854	<0.01
**OPL**	24572.39	24626.02	0.860	<0.01
**ONL**	21226.59	21264.64	0.865	<0.01
**PR**	31383.70	31369.14	0.941	<0.01
**RPE**	34760.16	34729.14	0.895	<0.01
**Whole scan area**	19244.68	19261.39	0.865	<0.01

Image Q: image quality score; ICC: intraclass correlation coefficient; RNFL: retinal nerve fiber layer; GCL: ganglion cell layer; IPL: inner plexiform layer; INL: inner nuclear layer; OPL: outer plexiform layer; ONL: outer nuclear layer; PR: photoreceptor; RPE: retinal pigment epithelium.

**Fig 3:**
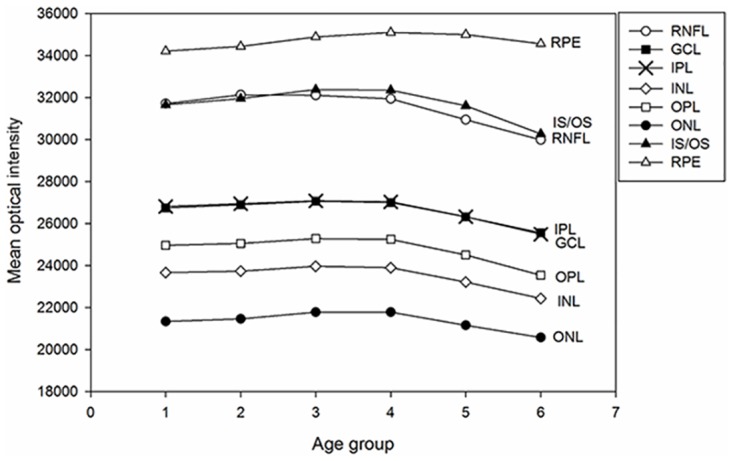
Mean optical intensity of retinal layers in different age groups. Groups 1 to 6 represent the age groups (shown in Table 1). Mean optical intensity of different retinal layers are shown in corresponding legends. Mean optical intensity was highest in RPE layer, followed by photoreceptor layer, RNFL, IPL, GCL, OPL, INL and ONL. Optical intensity was stable prior to 50 years of age, and then decreased in most retinal layers (RNFL to photoreceptor layer).

Table 4 shows the mean and SD of the macular retinal optical intensity of each age group. Mean optical intensity was highest in RPE layer, photoreceptor layer and RNFL, followed by IPL and GCL, and lowest in ONL (Fig 3). Optical intensity from RNFL to photoreceptor layer decreased with age after 50 years old (r ranged from -0.440 to -0.158, all P <0.01, Spearman’s test). For RPE layer, the optical intensity increased with older age groups (r = 0.318, P<0.01, Spearman’s test).

**Table 4:** Macular retinal optical intensity of different age groups.

Age (y)	20–29	30–39	40–49	50–59	60–69	70+	Total
**RNFL**	31716.53±1002.41	32130.35±536.43	32112.86±771.76	31945.08±806.99	30949.73±787.94	29987.39±1033.03	31558.68±1097.91
**GCL**	26745.71±671.09	26904.80±569.90	27065.58±778.21	27009.75±805.48	26323.79±929.56	25561.10±838.19	26653.71±896.17
**IPL**	26822.60±670.00	26949.93±569.32	27087.39±767.53	27040.68±754.32	26327.70±918.01	25492.97±823.89	26677.85±903.37
**INL**	23667.89±644.00	23734.76±561.02	23969.33±752.29	23898.58±733.82	23215.58±939.54	22439.48±810.12	23538.66±875.85
**OPL**	24961.40±678.86	25052.67±600.28	25284.47±768.46	25247.65±721.44	24506.97±979.62	23542.35±814.52	24824.14±933.38
**ONL**	21340.80±572.63	21464.02±536.60	21784.25±691.05	21781.30±656.26	21155.73±835.27	20576.29±955.45	21381.69±794.03
**PR**	31660.07±1404.50	31955.85±1359.32	32386.75±1617.76	32353.75±1055.47	31614.73±1009.89	30272.35±1194.85	31759.63±1444.96
**RPE**	34213.64±677.90	34438.35±515.59	34888.06±632.49	35103.28±581.10	35002.94±690.66	34563.16±623.89	34677.2±695.81

RNFL: retinal nerve fiber layer; GCL: ganglion cell layer; IPL: inner plexiform layer; INL: inner nuclear layer; OPL: outer plexiform layer;

ONL: outer nuclear layer; PR: photoreceptor layer; RPE: retinal pigment epithelium.

**Fig 4:**
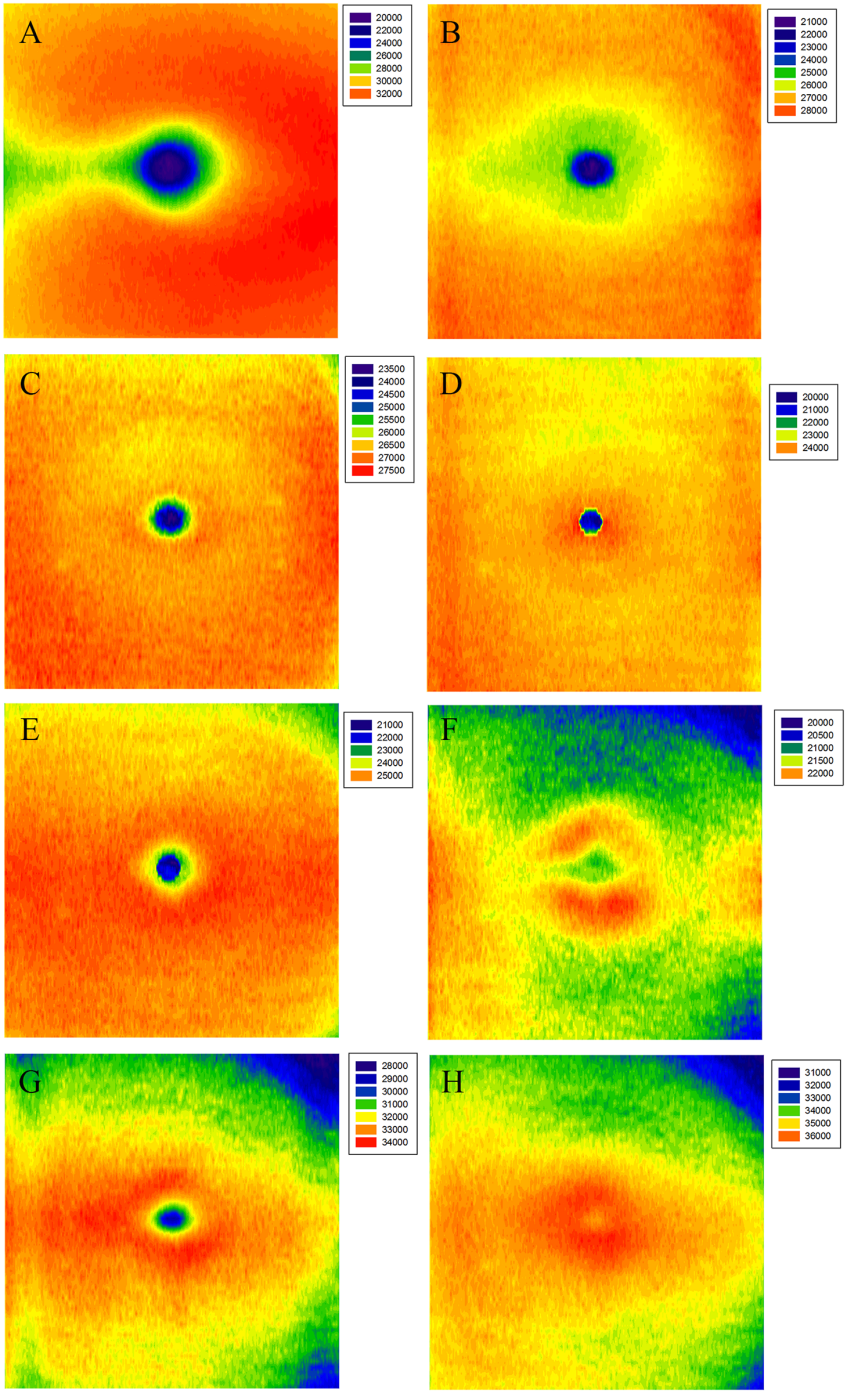
Mean optical intensity distribution of retinal layers. Color spectra to the right of each image show the optical intensity range of each layer. The right side of each image represents the nasal quadrant, the left side represents the temporal quadrant. The retinal layer is stated in the upper left of images. (A) RNFL, (B) GCL, (C) IPL, (D) INL, (E) OPL, (F) ONL, (G) photoreceptor, and (H) RPE.

Mean optical intensity for all subjects by ETDRS region is shown in Table 5. Optical intensity maps (Fig 4) show specific distribution patterns. In the central area, the optical intensity of RNFL, GCL, IPL, INL, OPL and photoreceptor layer were at a lower level while that of RPE was at its highest. There was a circinate area with higher intensity at the parafoveal region in ONL. In RNFL and GCL, mean optical intensity of the nasal sectors was greater than the temporal sectors, while ONL, photoreceptor layer and RPE showed the opposite distribution (P <0.05, paired t-test).

**Table 5:** Macular optical intensity in ETDRS regions.

**A**					
**Retinal layers**	**Cen**	**Inner Ring**			
		**Sin**	**Nin**	**Iin**	**Tin**
**RNFL**	24102.52±1251	30843.87±1384	29839.42±1422	30687.22±1322	29005.43±1545
**RGCL**	25053.23±1124	26148.73±1131	25942.91±1061	25876.91±1091	25798.12±1064
**IPL**	25903.58±1137	26641.79±1126	26615.80±1064	26336.48±1096	26583.51±1055
**INL**	24052.05±1133	23625.45±1076	23624.06±1012	23300.45±1047	23619.90±1006
**OPL**	24023.62±1217	25261.45±1120	25256.25±1038	24893.74±1094	25194.93±1093
**ONL**	21486.65±975	21807.71±1015	21652.77±961	21569.35±1013	21617.72±971
**PR**	31574.06±1734	32899.26±1859	32778.81±2176	32590.06±2165	33227.47±1839
**RPE**	36030.78±1070	35443.49±1003	35489.23±1103	35060.54±1218	35672.81±983
**B**					
**Retinal layers**	**Outer Ring**				**Whole ETDRS**
	**Sout**	**Nout**	**Iout**	**Tout**	
**RNFL**	32285.48±1480	32742.87±1521	31618.63±1231	29972.42±1564	31558.68±1098
**RGCL**	27122.16±1257	26911.89±1121	26825.53±1040	26494.2±1156	26653.71±896
**IPL**	26852.89±1275	26799.34±1111	26397.12±1032	26788.47±1141	26677.85±903
**INL**	23603.41±1178	23563.84±1039	23141.81±963	23692.28±1064	23538.66±876
**OPL**	24926.75±1242	25050.16±1084	24343.63±1057	25121.60±1125	24824.14±933
**ONL**	21377.63±1029	21406.76±944	20987.83±882	21605.05±943	21381.69±794
**PR**	31920.69±1905	31902.14±2123	31221.00±1843	32614.52±1723	31759.63±1445
**RPE**	34686.48±1048	34707.6±1102	33936.19±1058	35206.9±900	34677.20±696

Cen, central subfield; Sin, superior inner ring; Nin, nasal inner ring; Iin, inferior inner ring; Tin, temporal inner ring; Sout, superior outer ring; Nout, nasal outer ring; Iout, inferior outer ring; Tout, temporal outer ring; RNFL: retinal nerve fiber layer; GCL: ganglion cell layer; IPL: inner plexiform layer; INL: inner nuclear layer; OPL: outer plexiform layer; ONL: outer nuclear layer; PR: photoreceptor layer; RPE: retinal pigment epithelium.

### Determinants of retinal optical intensity

Differences between men and women in mean optical intensity were not significant (all P<0.05, independent-samples t-test). Table 6 shows the results of Pearson correlation analysis between optical intensity and age, height, weight, spherical equivalent, axial length, image quality, disc area and rim/disc area ratio. Image quality was significantly correlated with all retinal layer optical intensities, with the correlation coefficient r ranging from 0.503 to 0.851 (all P<0.01). Age was negatively correlated with optical intensity from RNFL to photoreceptor layer (-0.517<r<-0.242, P<0.01), but positively correlated with that in RPE (r = 0.287, P<0.01). Axial length was also negatively correlated with optical intensity from OPL to RPE. Spherical equivalent was positively correlated with only ONL and RPE optical intensity. The relationship of disc area and optical intensity in most layers was weak but significant in RPE layer. No statistically significant relationships were found between retinal optical intensity and height, weight and rim/disc area ratio.

**Table 6:** Correlation coefficients of retinal optical intensity with potential associated factors (univariate analysis).

	RNFL	RGCL	IPL	INL	OPL	ONL	PR	RPE
**Age**	-0.517**	-0.380**	-0.419**	-0.390**	-0.414**	-0.248**	-0.242**	0.287**
**Height**	0.014	0.05	0.033	0.030	0.045	-0.019	0.011	-0.040
**Weight**	-0.041	0.023	-0.005	0.001	0.013	0.010	0.013	0.127
**SE**	-0.104	-0.048	-0.046	0.004	0.037	0.147*	0.080	0.371**
**Axial Length**	-0.092	-0.066	-0.087	-0.126	-0.163*	-0.265**	-0.215**	-0.392**
**M image Q**	0.673**	0.753**	0.792**	0.831**	0.834**	0.851**	0.553**	0.503**
**Disc Area**	-0.024	-0.006	0.013	0.032	0.054	0.117	0.064	0.152*
**RD Area Ratio**	0.092	0.077	0.086	0.067	0.086	0.044	-0.036	-0.126

RNFL: retinal nerve fiber layer; GCL: ganglion cell layer; IPL: inner plexiform layer; INL: inner nuclear layer; OPL: outer plexiform layer; ONL: outer nuclear layer; PR: photoreceptor; RPE: retinal pigment epithelium;

** p<0.01;

*p<0.05.

Table 7 shows results of stepwise multiple linear regression analysis. Factors significant at P<0.05 with any retinal layer optical intensity from the univariate analysis were included. Significant determinants of optical intensity for most retinal layers were age and image quality. The effect of image quality was more pronounced in the optical intensity of ONL (β = 0.851), followed by INL, OPL and IPL, and was less pronounced in photoreceptor layer. The negative correlation between age and optical intensity from RNFL to OPL, except for ONL and photoreceptor layer, as well as the positive correlation between age and RPE optical intensity, remained after adjustment of other factors, such as image quality. The strongest association with age was the optical intensity of RPE layer (had the highest standardized β values = 0.456). No correlation was found, after adjustment, between retinal optical intensity and sex, height, weight, SE, axial length, disc area and rim/disc area ratio.

**Table 7:** Standardized coefficients in stepwise multiple regression analysis for retinal optical intensity.

	RNFL	RGCL	IPL	INL	OPL	ONL	PR	RPE
**Age**	-0.362**	-0.191**	-0.222**	-0.179**	-0.204**	—	—	0.456**
**SE**	N/A	N/A	N/A	N/A	N/A	—	N/A	—
**Axial Length**	N/A	N/A	N/A	N/A	—	—	—	—
**image Q**	0.576**	0.702**	0.732**	0.783**	0.779**	0.851**	0.553**	0.626**
**Disc Area**	N/A	N/A	N/A	N/A	N/A	N/A	N/A	—
**R**^**2**^	0.575	0.601	0.673	0.720	0734	0.724	0.306	0.446

RNFL: retinal nerve fiber layer; GCL: ganglion cell layer; IPL: inner plexiform layer; INL: inner nuclear layer; OPL: outer plexiform layer; ONL: outer nuclear layer; PR: photoreceptor; RPE: retinal pigment epithelium;—: exclude in stepwise regression;

**p <0.01.

## Discussion

In this study, by analyzing three-dimensional OCT data with our automatic software, we described the retinal optical intensity of adults in different age groups, and explored the effects of various factors on this parameter. The interoperator reproducibility of optical intensity measurement in healthy eyes was good. Mean optical intensity was highest in RPE layer, photoreceptor layer and RNFL, and lowest in ONL. Optical intensity was low in the central area of RNFL, GCL, IPL, INL, OPL and photoreceptor layers, and high in the center of RPE layer. In RNFL and GCL, mean optical intensity of the nasal sectors was greater than in the temporal sectors, whereas the ONL, photoreceptor layer and RPE had the opposite distribution. Our results also demonstrated that retinal optical intensity of most retinal layers increased with image quality, and decreased with age. There was no relationship between retinal optical intensity and sex, height, weight, SE, axial length, disc area or rim/disc area ratio.

### Mean retinal layer optical intensity

Reproducibility is critical for all imaging systems. In our study, under standard operating procedures, the interoperator ICCs for mean optical intensity ranged from 0.815 to 0.941, indicating that standard operation for OCT scanning allows reliable measurement of retinal optical intensity.

Consistent with hyperreflective bands in the OCT image, the mean optical intensity of RPE, photoreceptor layer and RNFL were the highest, consistent with our previous results [28], as well as with the light reflection profiles with OCT [29, 30]. Stronger subcellular reflectivity material, denser arrangement and more proper scan angle may account for the high optical intensity in these layers. Prior results [31] indicated that melanin granules were a primary OCT correlate for high reflectivity in RPE layer [31]. Furthermore, melanosomes in RPE cells were considered as a candidate OCT reflectivity source [32, 33]. In regard to the photoreceptor layer, mitochondria were thought to be the main contributors to reflectivity [30, 34]. The photoreceptor inner segment oval body contains an abundance of mitochondria, which have been proven to have a high refractive index [34]. In fact, the outer segment, which lacks mitochondria, was relatively hyporeflective (Fig 2), consistent with a prior publication concerning OCT segmentation [35]. In our study, the IS and OS were combined together into the photoreceptor layer for analysis. Therefore, optical intensity of photoreceptor layer was high as a whole. Because of the cylindrical nature and parallel arrangement of retinal nerve fiber or plexiform structure, reflectivity is expected to depend highly on the incident angle of the light. Usually, the measurement beam is perpendicular to the macula during a standard OCT scan. Thus, reflection of this structure will be higher than structures that consist of cell bodies, such as INL and ONL [36], which may produce isotropic scattering rather than reflection. This would also explain why reflectivity of RNFL decreases rapidly at the optic disc margin where the nerve fiber descends perpendicularly into the optic nerve. However, we also found that the optical intensity of GCL and IPL at different ages were almost identical and higher than that in OPL, which differed from the common view that plexiform layer may have higher reflectivity without quantitative assessment [36]. We considered that the optical intensity of certain retinal layers would be affected by the overlying layers, since the scan beam needs to pass through the superficial retinal layers before reaching the deeper layers, leading to a relative “decrease” in optical intensity of plexiform layers. This result may explain why these two layers are difficult to distinguish from each other by current commercially available OCT devices. However, by determining their differences in distribution pattern, we were able to separate the layers. Characteristic arrangement patterns of Henle fibers in OPL may be another reason for the decrease of optical intensity in OPL. The standard OCT imaging method, where light is directed perpendicular to the retina, fails to produce the maximum reflection of Henle fibers, since these fibers run obliquely and backscatter light in the direction that beyond the detection axis. Only after adjustment of scan angle can Henle fibers be well visualized [37, 38]. Therefore, in our study, the OPL referred to only the inner one third (photoreceptor synapse), while the outer two thirds were included in ONL.

### Regional distribution of retinal layer optical intensity

Quantifying RNFL damage is a well-developed method in glaucoma diagnosis [39]. Location of RNFL optical intensity defects may be correlated with glaucoma severity, as shown in a study by van der Schoot et al [19]. Since essential pathologic changes of glaucoma involves injury to ganglion cells and their axons, assessment of GCL/IPL has also been applied and proven to be of value, comparable to that of RNFL analysis, for the early diagnosis of glaucoma [40, 41]. Therefore, knowing the distribution of retinal layer optical intensity is important. In the control group of a study of by Barthelmes et al [8], reflectivity of the macular RNFL in the central area was the lowest compared to other regions, and the nasal sector showed a higher reflectivity than in the temporal sector. We confirmed these results and further suggested that this was consistent with the anatomical arrangement pattern of RNFL, where the nerve fibers become much more compact near the optic nerve head. Regarding the GCL, the optical intensity distribution of ganglion cells was consisted with previous histological findings in adults [42]. The highest ganglion cell density was 32000–38000 cells/mm^2^, lying in an elliptical area extending 0.4 to 2.0 mm from the fovea (corresponding to the ETDRS inner ring and inner part of the outer ring). Meanwhile, the density of the nasal sector was much higher by 300% than that in the temporal sector, and the superior sector density was higher by 60% than that in the inferior sector in the area between 2.0 to 4.0 mm from the fovea, which may explain the regional difference in optical intensity of GCL. Since IPL is composed of synapses and dendrites of bipolar cells, amacrine cells and ganglion cells, the fact that IPL optical intensity distribution was not identical with that of GCL was understandable.

Previous histological studies showed that ONL has a 10-layer cell nucleus at the fovea, the number of which decreases towards the periphery to 4 layers near the temporal side of the optic disc. Our results did not show a similar distribution in optical intensity (see Fig 4F), which may be due to our incorporating Henle fibers into ONL. The optical intensity of photoreceptor layer was lowest at the central area and higher at the peripheral region, in which was the opposite of RPE layer. The difference in optical intensity distribution between the two layers can be explained, based on previous histological studies [30–33], by the variation in distribution of mitochondria and melanosome respectively. Unlike the orientation and arrangement of mitochondria in other cells, mitochondria in the inner segment ellipsoid are long, thin (~0.25 × 3 μm), and tightly arranged, and they increase in number and become more compact with increasing eccentricity. For RPE layer, there are more melanosomes in the cells of central area than in the peripheral region [32], which may be the main determinant for the regional distribution of RPE optical intensity.

### Determinants of retinal layer optical intensity

Previous histological studies have reported that ganglion cell loss occurred with increasing age [43, 44] at a rate of approximately 0.3% to 0.6% per year [45, 46], and may accelerate after middle age [47]. The number of age-related loss of nerve fibers reaches 4000 to 5000, and may account for 35% of the loss in total nerve fibers [48, 49]. For INL, the number of bipolar cells decreases with age [50], especially after one’s 40s. Similarly, after adjustment for image quality (excluding the effect of cataract), our study found that optical intensity of RNFL, GCL, IPL and INL decreased with age. Therefore, age should be taken into consideration in disease diagnosis to distinguish from pathologic changes.

Studies listed above [43, 44] also found that the number of photoreceptors showed age-related loss. However, a study by Curcio et al found that foveal rods decreased with age, whereas cones did not [51]. Thus, photoreceptor optical intensity may remain stable with age because mitochondria, which are the source of reflectivity, are fewer in rods and more in cones [52]. Gao et al also determined that the density of the peripheral retinal pigmented epithelium cells decreased with age, whereas the density in the macular region was almost identical in age groups from the 20s to 90s [43]. In our study, we found that the optical intensity of RPE layer increased with age. We considered that because of cell aging, although the number of melanosomes decreases [53], lipofuscin in the cells may accumulate and combine with melanin to enlarge melanosomes [54] and increase the total reflectivity, despite a lack of change in cell density. This result is consistent with a prior finding using spectrum analysis [55].

Since the OCT signal comes from the reflective beam of retina, reflectivity may be affected by media opacity [29] that measuring beam passing thought and superficial tissue of target layer [56]. Normalization is therefore needed with respect to these interference. Our results indicated that retinal layer optical intensity correlated with image quality, suggesting that image quality can be used as a reference for normalization of optical intensity, as it represents the signal strength of the OCT machine and is directly provided by the OCT devices. Previous studies have used optical intensity of RPE layer [13, 19], vitreous [57] and RNFL layer [11] by either taking a ratio or self-establishing a formula. However, according to our previous study, the tissue layers listed above vary in a population. In fact, the ONL layer may be a better reference due to less variation and higher correlation with other layers[28].

Based on the multiple regression analysis in our study, there was no significant relationship between retinal layer optical intensity and sex, height, weight, spherical equivalent, disc area and rim/disc area ratio. Although some variables listed above have been proven to be correlated with retinal layer thickness [20, 22, 58], because thickness and optical intensity represent different characteristics of material, the determinants may be different.

### Limitations

As a cross-sectional study, our study has a few limitations. There are fewer than 50 subjects in each age group. As a hospital-based, but not population-based study, our results might not represent the general population. Further investigation, including larger sample size and subjects from different areas is needed to build up a normative database. Furthermore, because of limitations of current 3D optical intensity analysis techniques, we only analyzed the retinal optical intensity of the macular region. Our results were less representative in glaucoma diagnosis. Further study on regional 3D RNFL optical intensity analysis near the optic nerve head is still needed. Moreover, the reflectivity of tissue optical intensity is affected by image quality. Selection of high quality scans (image quality > 45) and denoising processes in our analysis are rough methods to reduce the influence of image noise. In further studies, we will build a more powerful model to clarify the actual retinal layer optical intensity.

In conclusion, we investigated the three dimensional retinal optical intensity profile with our automatic algorithm. Mean optical intensity was highest in RPE layer, photoreceptor layer and RNFL, and lowest in ONL. For most retinal layers, the optical intensity was low in the central fovea, except for RPE layer. No difference in optical intensity was found between males and females. The optical intensity decreased with age from RNFL to OPL, and increased with age in RPE. Optical intensity may represent age-related changes of retinal tissue, indicating the effect of age needs to be taken into consideration when using OCT for diagnosis. In normal subjects, the optical intensity was affected by image quality, and normalization is needed when comparing optical intensity between different subjects.
